# Development of a work of breathing scale and monitoring need of intubation in COVID-19 pneumonia

**DOI:** 10.1186/s13054-020-03176-y

**Published:** 2020-07-31

**Authors:** Mylene Apigo, Jeffrey Schechtman, Nyembezi Dhliwayo, Mohammed Al Tameemi, Raúl J. Gazmuri

**Affiliations:** 1 Section of Critical Care Medicine, Captain James A. Lovell Federal Health Care Center, 3001 Green Bay Road, North Chicago, 60064 IL USA; 2 Department of Clinical Sciences, Internal Medicine Residency Program at Rosalind Franklin University of Medicine and Science, 3333 Green Bay Road, North Chicago, 60064 IL USA; 3 Department of Clinical Sciences, and Discipline of Physiology and Biophysics, Resuscitation Institute, Rosalind Franklin University of Medicine and Science, 3333 Green Bay Road, North Chicago, IL 60064 USA

COVID-19 pneumonia presents in most patients with scattered areas of lung involvement within healthy lungs displaying hypoxemia and tachypnea but with relatively minor reductions in lung compliance [1, 2]. Noninvasive ventilation and high-flow nasal cannula (HFNC) are reasonable initial interventions reserving endotracheal intubation for worsening disease severity evidenced by increased work of breathing (WOB), risking respiratory muscle fatigue leading to hypoventilation, hypoxemia, and cardiac arrest and large transpulmonary pressure swings risking patient self-inflicted lung injury (SILI) [3, 4].

Experts have suggested use of esophageal manometry (as surrogate of pleural pressure) and consider intubation when pressure swings exceeds 15 cm H_2_O identifying risk of SILI [5]. However, monitoring esophageal manometry in non-intubated patients is not a practical option. We previously developed a noninvasive WOB scale ranging from 1 to 7 based on respiratory physiology, combining the respiratory rate with use of respiratory accessory muscles (Fig. 1).

**Fig. 1:**
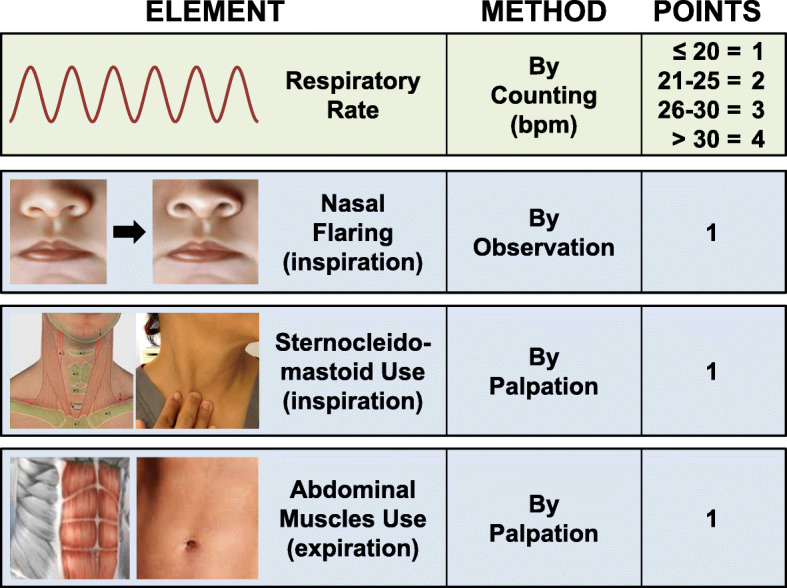
Work of breathing scale assigning points to the respiratory frequency and activation of respiratory accessory muscles. Nasal flaring is determined visually by noticing widening of the nostrils during inspiration while standing at approximately one-meter from the patient. Activation of the sternocleidomastoid is determined by gentle palpation of its clavicular insertion using two fingers from the hand ipsilateral to the patient’s side noticing increased tension during inspiration. Activation of abdominal muscles is determined by gentle palpation of the abdomen using the hand ipsilateral to the patient’s side noticing increased tension during expiration

We tested the ability of healthcare providers to rapidly learn and apply our WOB scale. We first trained a team of “super-raters” composed of ICU nurses and internal medicine residents. Next, we identified a group of nurses, medical students, residents, and attendings naïve to the WOB scale and designated them as “raters”. Super-raters trained raters using a 4-min WOB scale video and tested their ability to correctly rate the WOB level in 80 non-intubated patients from the Emergency Department, medical wards, and the ICU with WOB ranging from 1 to 5. A total of three assessments per patient were completed showing a high correlation between the super-raters and raters 1 (*r* = 0.93; *p* < 0.001), super-raters and raters 2 (*r* = 0.91; *p* < 0.001), and between the two raters (*r* = 0.84; *p* < 0.001). In addition, the interrater reliability between the two raters measured by the Krippendorf’s *α* test was also high at 0.85 (95% CI, 0.78–0.91).

We then examined the relationship between the respiratory rate and activation of respiratory accessory muscles in 110 patients (by adding 30 patients to the original 80 patients). As shown in Fig. 2, there was a low incidence of accessory respiratory muscle use when the respiratory rate was ≤ 20, yet with increased respiratory rate, the use of accessory respiratory muscles proportionally increased.

**Fig. 2:**
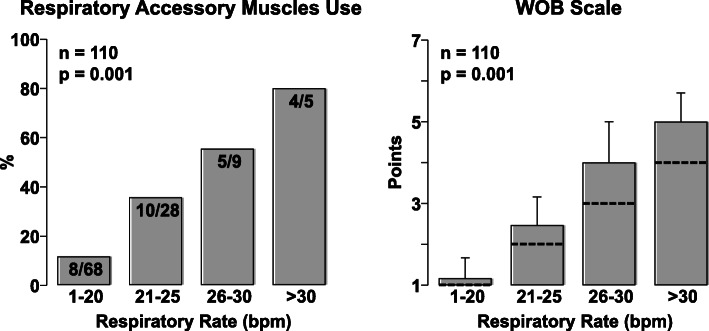
Left graph shows the percentage of patients who had activation of at least one of the accessory muscles assessed by the work breathing scale as a function of respiratory rate. Right graph shows the mean and standard deviation of the work breathing scale as a function of respiratory rate with the discontinuous line indicating the contribution of the respiratory rate alone (right). Analysis performed in 110 patients. Overall differences were analyzed using SigmaPlot 12.5 by chi-square on the left and by one-way analysis of variance on the right

We examined the performance of our WOB scale in 10 patients admitted to the ICU with radiographic evidence of extensive COVID-19 pneumonia, significant hypoxemia, and multiple risk factors associated with poor outcome. Their mean age was 63 years (95% CI 50 to 75) and stayed in the ICU for 8 days (95% CI 5 to 10). Nine patients received HFNC over 6 days (95% CI 3 to 8). The WOB level was measured every 4 h. The maximum WOB was 4.3 (95% CI 3.6 to 5.0), contributed primarily by respiratory rate with a score of 3.6 (95% CI 3.2 to 4.0) and infrequent use of respiratory accessory muscles. All 10 patients survived without need of intubation. For comparison, three other patients who needed intubation had a maximal work of breathing within the preceding 24 h of 5.3 (95% CI 2.5 to 8.2). The respiratory rate score was 3.8 (95% CI 2.2 to 5.1) similar to non-intubate patients but with more often use of respiratory accessory muscles.

Our data suggest that patients with COVID-19 pneumonia can be supported for extended periods using HFNC despite tachypnea provided there is only infrequent and modest use of respiratory accessory muscles, corresponding to a WOB scale ≤ 4, prompting closer assessment for possible intubation when WOB > 4. This approach would be especially advantageous under conditions of high disease intensity when avoidance of intubation is likely to result in a better outcome [6]. Further work in a larger cohort of patients is awaited.

## Data Availability

The dataset used for the current study is available from the corresponding author on reasonable request.
